# Congenital High Airway Obstruction Syndrome (CHAOS): No Intervention, No Survival—A Case Report and Literature Review

**DOI:** 10.1155/2020/1036073

**Published:** 2020-06-27

**Authors:** Ammar Ashraf, Ahmed Mohamed Abdelrahman, Ahmed Senna, Fatimah Alsaad

**Affiliations:** ^1^Department of Medical Imaging, King Abdulaziz Hospital, Ministry of National Guard Health Affairs, Al-Ahsa, Saudi Arabia; ^2^Department of Neonatology, King Abdulaziz Hospital, Ministry of National Guard Health Affairs, Al-Ahsa, Saudi Arabia; ^3^Department of Medical Imaging, King Fahad Hospital, Ministry of Health, Al-Ahsa, Saudi Arabia

## Abstract

Congenital high airway obstruction syndrome (CHAOS) is complete or partial obstruction of the fetal upper airway. CHAOS is a rare and fatal condition if no perinatal intervention is done. Antenatal sonographic imaging has typical findings that can help in an early diagnosis, which is important in deciding elective termination of the pregnancy or successful planning of appropriate perinatal management.

## 1. Introduction

Congenital high airway obstruction syndrome (CHAOS) is a rare life-threatening disorder caused by complete or near-complete obstruction of the fetal upper airway [1–7]. It was first described by Hedrick in the late 1900s [1, 2, 4, 8]. Laryngeal atresia or stenosis is the commonest cause of CHAOS [1, 2, 4, 7, 9]. CHAOS is usually a fatal condition that is not compatible with life and leads to a stillbirth or early neonatal death if not recognized in the antenatal period [1, 2, 4, 7]. Due to recent marked improvements in imaging techniques, more cases are being recognized in utero nowadays [1, 2, 4, 7]. Bilateral enlarged hyperechoic lungs, flattened or inverted leaflets of the diaphragm, and dilated fluid-filled tracheobronchial tree are the typical antenatal sonographic findings. Fetal ascites and nonimmune hydrops are the other associated findings of this syndrome [1–6, 10]. Accurate prenatal recognition of this lethal entity is becoming an important issue, as currently available management options, particularly, ex utero intrapartum treatment (EXIT), have a promising favorable neonatal outcome [1, 3, 4, 11, 12].

Here, we report a case of congenital high airway obstruction syndrome due to laryngeal atresia diagnosed on routine antenatal ultrasound examination at 18 weeks of gestation. We also review the relevant literature in brief.

## 2. Case Report

**Figure 1:**
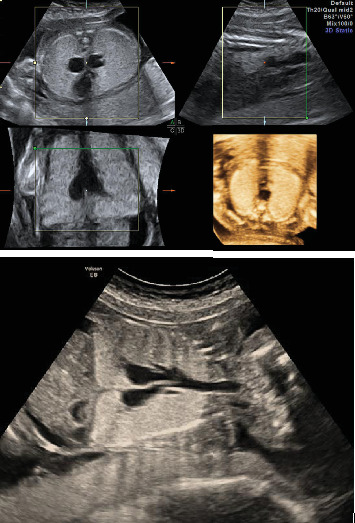
Enlarged echogenic lungs, dilated trachea, bronchial tree, and flattened diaphragm.

**Figure 2:**
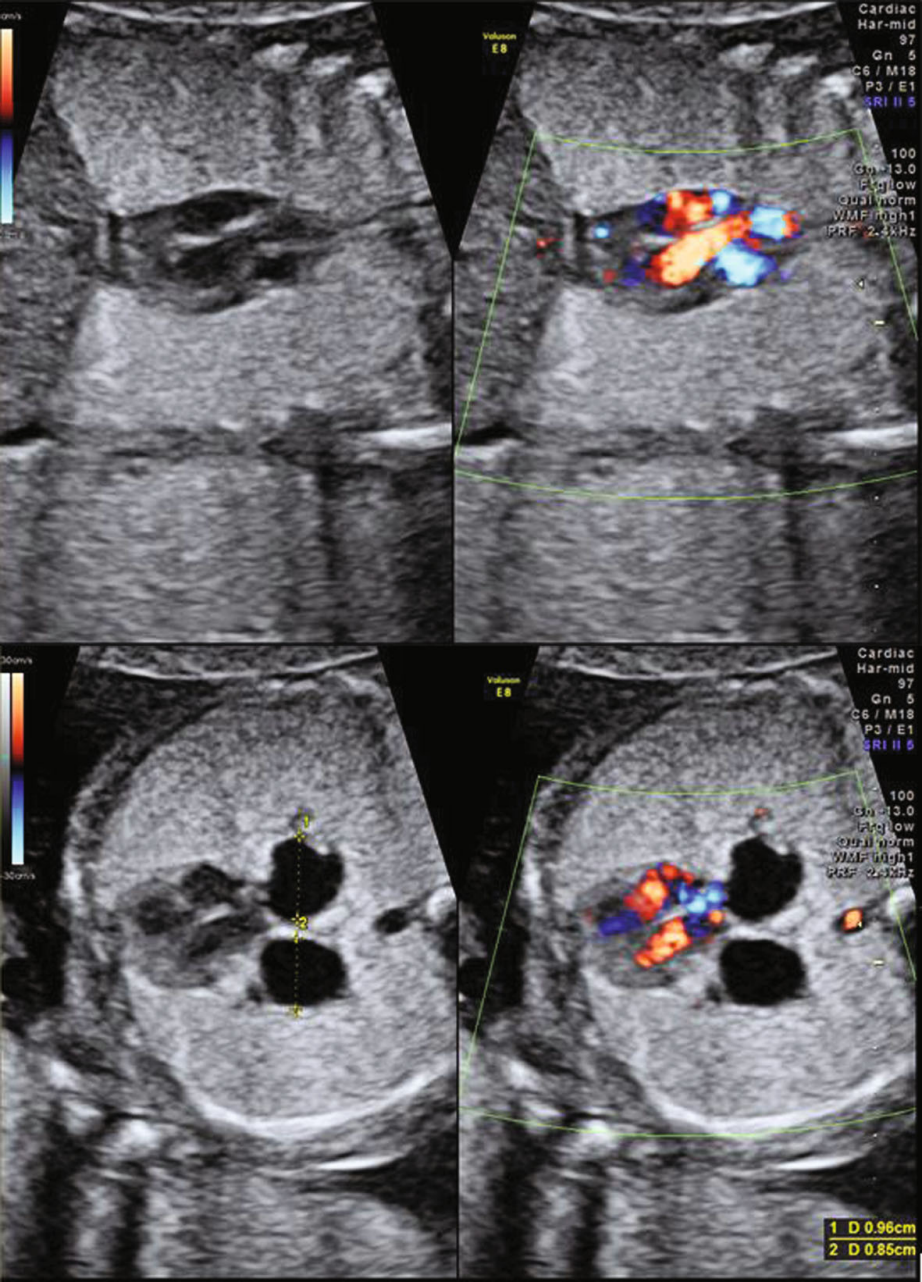
Small centrally placed and compressed heart by large echogenic lungs, dilated bronchi, and flattened diaphragm.

**Figure 3:**
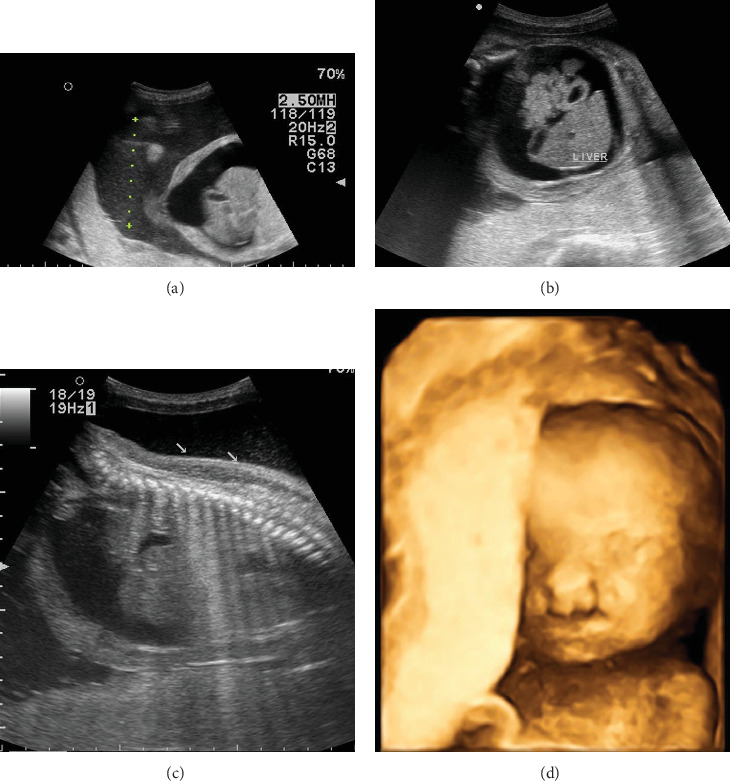
Polyhydramnios (a), ascites (b), subcutaneous edema (c), and cleft lip (d).

**Figure 4:**
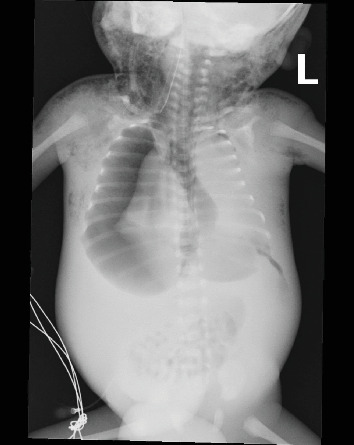
Chest radiograph frontal view. Large right pneumothorax, pneumomediastinum, and surgical emphysema in the neck and chest. Abnormally oriented/positioned endotracheal tube. The radiograph also showed nonaerated lungs, inverted diaphragm, abdominal distension, and thickened subcutaneous soft tissues.

30 years old (gravida 2, para 1) with a history of 18 weeks of pregnancy came to our department for a routine second-trimester antenatal ultrasound examination. In the family history, except for first-cousin marriage, there was no significant history predisposing to genetic/familial disorders. Ultrasound examination was performed, and it showed a single viable intrauterine fetus with bilateral enlarged echogenic lungs and dilated fluid-filled trachea. Small centrally displaced heart, flattened diaphragm, cleft lip, and cleft palate were also noted (Figures 1 and 2). No other abnormalities were seen. MRI was advised for further evaluation, particularly to see the exact level of airway obstruction. Diagnosis and its poor prognosis were discussed in detail with family. The family was also offered a referral to a higher specialist center where the facility of ex utero intrapartum treatment (EXIT) was available; however, unfortunately, parents refused further workup, any intervention as well as pregnancy termination. Two months later, she presented in the emergency department with preterm labor. Ultrasound examination was done which showed interval development of polyhydramnios, ascites, diffuse subcutaneous edema (nonimmune fetal hydrops), and mild cervical dilatation, in addition to redemonstration of previous sonographic findings (Figure 3). No omphalocele was detected on both ultrasound examinations. No gross cardiac abnormality was seen on limited cardiac views; however, no dedicated fetal echocardiography was performed. Parents again refused any intervention. Two days later, she delivered (spontaneous vaginal delivery) a 30 weeks preterm male baby with an average weight of 1700 grams. The baby was delivered alive with a fetal heart rate of 114 beats per minute but with no breathing. Immediate respiratory resuscitation with an Ambu bag was done. Laryngoscopy was also done which showed severe laryngeal atresia. Endotracheal intubation was attempted which was associated with significant airway trauma and was not successful. Chest radiograph (Figure 4) was obtained after the intubation, which showed large right pneumothorax, pneumomediastinum, and surgical emphysema in the chest and lower neck, which was likely related to attempted traumatic intubation. The radiograph also showed nonaerated lungs, inverted diaphragm, abdominal distension, and thickened subcutaneous soft tissues, which were consistent with the antenatal sonographic findings of fetal hydrops. The baby had dysmorphic facial features (cleft lip and cleft palate), omphalocele, abdominal distension, small genitalia with mild bilateral hydroceles, and generalized anasarca on clinical examination. Baby expired within two hours of birth. The family refused autopsy but opted for chromosomal analysis, to exclude any underlying associated genetic abnormality. Chromosomal analysis was requested which, unfortunately, could not be performed due to poor specimen preservation.

## 3. Discussion

Congenital high airway obstruction syndrome is a very rare entity with very poor prognosis in which proximal airway is completely or partially obstructed with laryngeal atresia being the most frequent cause 13. Its true incidence is unknown but may be higher than previously thought [1, 6–8].

More than 100 case reports and some case series of CHAOS had been reported in the literature [13].

Laryngeal/tracheal atresia is a rare congenital anomaly that takes place by deficient recanalization of the upper airways at 9–10 weeks of embryological development leading to a clinical spectrum defined as congenital high airway obstruction syndrome (CHAOS) [1–4, 8].

Normally, the fluid secreted by fetal lung is absorbed through the tracheobronchial tree [1, 4]. However, in the case of airway obstruction, this fluid cannot be appropriately drained and keeps on accumulating in the fetal lungs which results in a gradual rise in intratracheal pressure and leads to hyper expansion and abnormal development of the lungs [1–4]. The hyper expanded lungs compress the heart, great veins, and diaphragm. The heart displaces centrally and becomes small and dysfunctional. Dysfunctional cardiovascular system and decreased venous return lead to ascites and nonimmune hydrops. The diaphragm flattens or inverts depending on the severity of the process [1–4, 6, 7, 14–16]. These series of events are responsible for the prenatal imaging characteristics of CHAOS [2]. Tracheal dilatation is characteristic; however, the exact site of airway obstruction cannot be confidently visualized on antenatal sonography [3].

The main pathology is an intrinsic obstruction of the upper airway. Laryngeal atresia is the commonest cause. However, tracheal atresia/stenosis, laryngeal/subglottic stenosis, laryngeal webs, and laryngeal cysts are other possible causes of CHAOS [1–4, 8, 12, 14]. CHAOS can also be caused by extrinsic causes like cervical teratoma, lymphatic malformation, and vascular rings like double aortic arch [1, 8].

Though the majority of the cases of CHAOS are sporadic, approximately half of the cases of CHAOS are associated with congenital anomalies, especially of the skeletal system [1, 7, 8]. The commonest associated genetic disorder with CHAOS is Fraser syndrome, which is an autosomal recessive disorder characterized by laryngeal atresia, syndactyly, cryptophthalmos, and urogenital defects and can be confirmed by mutation analysis of FRAS1 [2–4, 7, 17]. Other syndromes reported in association with CHAOS are Cri-du-Chat syndrome, velocardiofacial syndrome, and short-rib polydactyly syndrome [1, 2, 4, 7]. Due to the possibility of coexistence of these genetic syndromes with CHAOS, all patients with CHAOS need detailed evaluation to avoid inheritance for future pregnancies [1, 4].

In our case, parents refused autopsy; however, they agreed for genetic/chromosomal analysis which, unfortunately, could not be performed due to technical reasons.

Imaging is used to document the typical radiological features of CHAOS and to investigate the cause and level of the airway obstruction; intrinsic causes (laryngeal versus tracheal) versus extrinsic causes (lymphatic malformations, cervical tumors, and vascular anomalies) [3, 4, 6, 8, 12, 15].

CHAOS can be diagnosed on routine antenatal ultrasound examination as early as 16 weeks 13. Ultrasound is the first-line imaging modality of choice in antenatal diagnosis of CHAOS due to its widespread and easy availability as well as its low cost [1, 4]. Ultrasound can demonstrate the dilated airways but has limitations in defining the precise level of obstruction (laryngeal versus tracheal) [7, 14]. MR imaging can be utilized as an additional imaging tool to define the exact level of obstruction, particularly if any operative intervention is planned in the future [1, 6, 7, 14, 18]. MRI is also superior to ultrasound in excluding extrinsic causes of laryngotracheal obstruction [1, 4, 7, 14].

Amniotic fluid volume is variable and depends on the gestational age of the fetus and other associated congenital anomalies/syndromes; for example, oligohydramnios is seen in early pregnancy and cases associated with Fraser's syndrome. Polyhydramnios (caused by compression of the esophagus by enlarged lungs and dilated airways) is seen in late pregnancy [1, 2, 4, 7].

Classical sonographic features of CHAOS are bilateral boggy hyperechoic lungs, fluid-filled dilated airways, small compressed and centrally displaced heart, flattened/inverted diaphragm, and ascites. All these sonographic features can also be demonstrated on MRI [1–4, 9, 10, 16].

Sometimes pulmonary enlargement may resolve spontaneously which may be secondary to spontaneous perforation of dilated airways or trachea-esophageal fistula [1, 2, 4, 6, 14]. It is important to remember that the apparent resolution of fetal hyperechoic lungs on ultrasound does not necessarily mean resolution of the underlying pathology [6].

Our case was diagnosed at 18 weeks of gestation and had characteristic sonographic features. Amniotic fluid amount was normal on initial scan; however, follow-up scan at 30 weeks of gestation showed interval development of polyhydramnios. Fetal ascites and generalized subcutaneous edema were also noted on follow-up scan. Depending on the degree of dilatation of the trachea, we assumed that the level of airway obstruction was in the upper airway, likely at the level of larynx, which was confirmed by laryngoscopy after delivery. Our patient also had cleft lip and cleft palate which were noted on both ultrasound examinations. We could not find any association of this anomaly with CHAOS in literature and failure of genetic analysis also did not help us in establishing/excluding such association. We presumed it was likely an incidental finding.

The differential diagnosis of CHAOS includes other common fetal hyperechogenic lung lesions like congenital cystic adenomatoid malformation (CCAM) and pulmonary sequestration. CCAM is usually unilateral (rarely bilateral) and can show spontaneous resolution during pregnancy. It can also be differentiated from CHAOS by tracheal enlargement which is specific for CHAOS. Pulmonary sequestration is differentiated from CHAOS based on systemic arterial supply [1, 2, 4, 6, 14].

## 4. Treatment

Previously, CHAOS was considered a lethal disease with a grave prognosis [1, 4]. However, recently, some infants with prenatally diagnosed CHAOS have been reported to be treated successfully by ex utero intrapartum treatment (EXIT) [3, 6]. Antenatal diagnosis of CHAOS is the key to any successful perinatal management [14]. Live born neonates with CHAOS usually have multiple postnatal problems including respiratory distress caused by the abnormal development of the lungs and diaphragmatic dysfunction [2]. The main purpose of any perinatal therapeutic intervention is to secure an intact airway before the baby is delivered [1, 4]. Ex utero intrapartum treatment (EXIT) can be performed in patients with incomplete obstruction, diagnosed in the late 2nd or 3rd trimester of pregnancy and with absent severe hydrops [1, 4, 14]. In this procedure, laryngoscopic examination followed by tracheostomy is done after partial delivery of the fetal head with the fetus still being attached to the placenta [14]. The other available treatment option is intrauterine fetoscopic laser laryngotomy which is based on the concept of spontaneous antenatal improvement of some cases of CHAOS secondary to spontaneous perforation [7, 14]. This procedure also does not affect the fetomaternal circulation but is associated with the risk of premature rupture of membranes (PROM) [14].

Although, the commonest cause of airway obstruction in CHAOS is laryngeal atresia; rarely isolated stenosis or web, which are usually associated with normal laryngeal anatomy, may be the cause of airway obstruction and release of such obstruction can ultimately restore normal lung development [16]. Fetoscopic wire tracheoplasty is an option in such selected cases of CHAOS with short-segment lesions and webs being more amenable than long segment lesions or complete agenesis of an airway segment [11].

Fetal MRI may help in patient's selection that can benefit from fetoscopic tracheoplasty by determining the level and approximate length of the obstruction [11]. Fetal lie, fetal head orientation, and placental location (i.e., posterior placenta or anterior placenta with a clear window for intervention) are important factors regarding this intervention [11].

In conclusion, congenital high airway obstruction syndrome is an uncommon cause of proximal airway obstruction which is not compatible with life if no perinatal intervention is done. Antenatal sonographic imaging has typical findings that can help in establishing an early and accurate diagnosis. MRI is used as an adjunct diagnostic tool, particularly in determining the level of airway obstruction and in excluding extrinsic causes of airway obstruction. MRI is also especially important if any perinatal fetal intervention is planned.
